# Diagnostic Scores and Treatment Options for Acute Disseminated Intravascular Coagulation in Children

**DOI:** 10.7759/cureus.17682

**Published:** 2021-09-03

**Authors:** Saru Kunwar, Mohammad Alam, Francis Ezekwueme, Muhammad Yasir, Jannel A Lawrence, Sunil Shah, Domonick K Gordon

**Affiliations:** 1 Internal Medicine/Pediatrics, California Institute of Behavioral Neurosciences & Psychology, California, USA; 2 Internal Medicine, California Institute of Behavioral Neurosciences & Psychology, California, USA; 3 Emergency Medicine, California Institute of Behavioral Neurosciences & Psychology, California, USA

**Keywords:** children, diagnosis, dic, disseminated intravascular coagulation, pediatric, scoring system, treatment

## Abstract

Disseminated intravascular coagulation (DIC) is a thrombo-hemorrhagic condition that commonly accompanies life-threatening illnesses in children and is associated with significant morbidity and mortality. Treatment of underlying conditions, hemodynamic support, and replacement therapy with blood components is the mainstay of DIC management. Limited research studies have supported the use of antithrombin (AT), recombinant thrombomodulin (rTM), and protein C concentrates (PrCC). Although there have been several studies and advancements in the DIC treatment in adults, data in pediatric patients are limited, and the consensus is lacking. Evidence validating the use of diagnostic scoring systems in the pediatric population is also limited. Since the hemostatic system differs significantly in children, especially in neonates, management of DIC is also different in children from that of adults, and there is a dire need for good quality research studies in this aspect.

We reviewed more than 100 articles in PubMed, Cochrane database, and Google Scholar. This traditional review article discusses different scoring systems for diagnosing DIC in pediatric patients, and different pharmacological treatment options for acute DIC in this population. This study mainly focuses on papers published from 1990 to 2021 and includes papers in all languages involving humans only.

## Introduction and background

Disseminated intravascular coagulation (DIC) is an acquired clinicopathological syndrome that complicates various illnesses, mainly sepsis, trauma, malignancy, liver diseases, and toxins. It is characterized by systemic activation of different coagulation pathways resulting in the generation of fibrin clots that may cause organ failure. In addition, there is concomitant consumption of platelets and coagulation factors, which may result in clinical bleeding [1].

The mainstay of management of DIC is early identification and treatment of the underlying condition, hemodynamic support, frequent monitoring of laboratory and clinical parameters, and replacement of consumed coagulation factors and blood components via transfusion of platelets, fresh frozen plasma (FFP), or cryoprecipitate. However, prophylactic transfusion of these blood products is not recommended unless there is a bleeding or impending invasive procedure [1,2]. Over the past few decades, several studies have demonstrated the efficacy of antithrombin (AT) and protein C concentrates (PrCC), recombinant activated protein C (APC), and recombinant thrombomodulin (rTM) for the management of DIC in children [2,3].

Although it is a common thrombo-hemorrhagic condition in critically ill children, there have been very limited studies and only very few controlled clinical trials regarding its management in pediatric patients [2,3]. In addition, several diagnostic scoring systems have been validated for calculating morbidity and mortality risks in adults but not in pediatric patients. The purpose of this traditional review is to briefly outline the diagnostic scoring systems and different pharmacological treatment options for acute DIC in children so as to guide pediatricians in their management. DIC due to congenital deficiencies of protein C, protein S, and antithrombin (AT), or treatments for chronic DIC or snake or scorpion bites are not included in this review.

## Review

Diagnostic scores for DIC

Several studies have proven the validity of the Japanese Association for Acute Medicine (JAAM), the Japanese Ministry of Health, Labor and Welfare (JMHLW), and the International Society on Thrombosis and Hemostasis (ISTH) DIC scoring systems in adults (details in Table 1) [4,5]. But there have been limited studies in pediatric patients, and these limited data suggest that these scoring systems perform reasonably well in pediatric patients [6-8].

**Table 1:** Comparison of different scoring systems for the diagnosis of DIC. DIC, disseminated intravascular coagulation; FDP, fibrin degradation products; FEU, fibrinogen equivalent unit; INR, international normalized ratio; ISTH, International Society on Thrombosis and Hemostasis; JAAM, Japanese Association for Acute Medicine; JMHLW, Japanese Ministry of Health, Labor and Welfare; PT, prothrombin time; SIRS, systemic inflammatory response syndrome; TCH, Texas Children’s Hospital.

	JAAM		ISTH		JMHLW		TCH values	
		Score		Score		Score		Score
Underlying disease	Essential	0	Essential		Essential	1		
	SIRS criteria met: ≥3	1			Organ failure	1		
	SIRS 0-2	0			Bleeding	1		
Platelet count (×10³ µL)	<80 or >50% reduction in 24 hours	3	<50	2	<50	3	Sequential measurement	
	81-120 or 30-50% reduction	1	50-100	1	50-80	2		
	≥120	0	>100	0	80-120	1		
PT/INR	INR ≥ 1.2	1	PT > 6 seconds	2	INR > 1.67	2	PT > 5.6 seconds	2
	INR < 1.2	0	PT 3-6 seconds	1	INR 1.25–1.67	1	2.6-5.6 seconds	1
			PT < 3 seconds	0			< 2.6 seconds	0
Fibrinogen (mg/dL)	<35	1	<100	1	<1	2	Sequential measurement	
	≥35	0	>100	0	1-1.5	1		
FDP (mcg/mL) or fibrin related markers	FDP ≥ 25	3	Strong increase	3	>40	3	D-dimer, FEU: 4	3
	10-24	1	Moderate increase	2	20-40	2	1.5-3.9	2
	<10	0	No increase	0	10-20	1	<1.5	0
Score interpretation	≥4: DIC diagnosis		≥5: overt DIC daily scoring; <5: suggestive for non-overt DIC, repeat in 1-2 days		≥7: DIC diagnosis			≥5: overt DIC

A retrospective analysis in 132 children found that a one-point rise in DIC score was associated with an increased risk of mortality [6]. Another similar retrospective study in 191 pediatric intensive care unit (PICU) patients revealed that the JAAM and ISTH scores were useful for detecting DIC in PICU patients. These scores correlated well with other severity scores: Pediatric Risk of Mortality III, modified Sequential Organ Failure Assessment, and Pediatric Multiple Organ Dysfunction Syndrome scores. The diagnostic concordance rate between ISTH and JAAM scoring systems was 52.6%. The scores were also significantly associated with 28-day mortality rates, and the areas under the receiver operating characteristic curve of JAAM score was (95% CI, 0.675-0.900) and ISTH score was 0.716 (95% CI, 0.598-0.834) [8].

The other diagnostic criteria for DIC are Texas Children's Hospital (TCH) criteria [7]. It does not have a scoring system, but the coagulation parameters (prothrombin time [PT], platelet count, fibrinogen, and D-dimer) and the patient's clinical condition are serially evaluated, and DIC is diagnosed when there is a successive drop or increment in the values resulting in a trend. Soundar et al. did a retrospective study of 130 children with DIC and evaluated the ISTH criteria and TCH criteria against the gold standard diagnostic method of autopsy in those who died [7]. They found that the TCH diagnostic criteria were comparable to the ISTH scoring system, or even better with significantly higher sensitivity (P < 0.05) when tested against the gold standard. They also concluded that fibrinogen is not a significant predictor of overt DIC, and also, sequential testing of coagulation parameters is recommended for better sensitivity while using the ISTH scoring system in pediatric patients [7].

The JMHLW scoring system has moderate sensitivity and high specificity for diagnosing DIC in hematologic malignancies in adults but there is not enough evidence to support its usefulness in critically ill patients [5]. Several studies, especially in Japan, have used the JMHLW scoring system to diagnose DIC in pediatric patients, but no comparative studies or mortality/morbidity risk assessment studies have been done [3,7,9].

Selecting a scoring system depends upon its use. Early diagnosis requires a scoring system with higher sensitivity, whereas a highly specific system is preferred to confirm the diagnosis [5]. Unfortunately, good quality studies are still lacking in the pediatric population that validate these scoring systems and guide the selection. However, it can be concluded that a one-time score should not be relied upon, but rather serial evaluation should be done for the definite diagnosis, to rule out DIC, and analyze the course and severity of DIC.

Basic principles of DIC management

Rajagopal et al. [2] summarized it as early identification and management of the underlying condition predisposing to DIC, supportive management with blood products and related measures, inhibiting the effects of excess thrombin, regular monitoring of clinical and laboratory parameters (keeping in mind that deranged values could be due to several factors, not just DIC), and early intervention from a multidisciplinary team.

Treatment of underlying disease

DIC never occurs in isolation, and early identification and vigorous management of the underlying condition is the cornerstone of DIC management. Therefore, it should always precede interventions to normalize coagulation parameters [10], e.g., empiric broad-spectrum intravenous therapy with one or more antimicrobials (bacterial and potentially fungal or viral coverage) as soon as possible after recognition and within one hour for those with sepsis or septic shock [11], and anti-snake venom for snake bites. However, in overt DIC, coagulopathy may persist even after the underlying primary cause of injury to endothelium is eliminated and may require specific therapy [10].

Wada et al. compared four DIC guidelines and recommends that treatment of underlying disorder should be attempted first in patients with bleeding, organ failure, and non-symptomatic types of DIC, while blood transfusions are needed first in patients with the massive bleeding type of DIC [12].

The common causes of DIC in neonates, older infants, and children are listed in Table 2 as described by Rajagopal et al. [2].

**Table 2:** Common causes of disseminated intravascular coagulation in neonates, older infants, and children. As described by Rajagopal et al. [2].

Causes	
Sepsis	Bacterial (group B streptococcus, *Neisseria meningitidis*, *Haemophilus influenzae*), viral (Cytomegalovirus, Varicella-zoster), fungal (systemic candidiasis, aspergillosis), and others like dengue and malaria
Perinatal	Birth asphyxia, respiratory distress syndrome, meconium aspiration syndrome, and dead twin
Injury	Trauma, burn, and drowning
Malignancy	Acute lymphoblastic leukemia, acute promyelocytic leukemia, and solid tumors
Others	Snake and spider bites, liver diseases, acute hemolytic transfusion reaction, and giant hemangioma (Kasabach-Merritt syndrome)

Management of shock

The first step is to maintain or restore airway, oxygenation, and ventilation followed by hemodynamic resuscitation. Davis et al. suggested the therapeutic endpoints of hemodynamic resuscitation in children as capillary refill less than or equal to two seconds; threshold heart rate; warm extremities and normal pulses (quality of the peripheral pulses equal to central pulses); urine output >1 mL/kg/hr; normal mental status; cardiac index 3.3-6.0 L/min/m2, with normal perfusion pressure for age; central venous oxygen saturation greater than 70%; normal INR, anion gap, and lactate; systolic blood pressure at least fifth percentile for age i.e. 60 mmHg for less than one month of age, 70 mmHg + [2 x age in years] for 1 to 10 years, and 90 mmHg for more than or equal to 10 years of age [13].

Replacement therapy

The commonly used blood components in DIC treatment include platelets, fresh frozen plasma (FFP), cryoprecipitate, and fibrinogen concentrates. However, since DIC is a procoagulant process, they should not primarily be administered based on laboratory results but should be administered only in patients with bleeding manifestations [2,14-16].

The recommended guideline for platelet transfusion is bleeding patients with platelet count <50,000/μL, but bleeding in DIC can also be due to platelet dysfunction, and patients with higher platelet count may also require platelet transfusion [14]. In non-bleeding patients, prophylactic platelet transfusion is not recommended as factors beyond platelet counts alone may impact bleeding risk in children. Also, thrombocytopenia is commonly seen in sick preterm neonates, and studies have shown that pediatric patients are at a higher risk of bleeding over a wider range of platelet counts [2,15]. For older or larger children, the dose is 4-5 U of platelets, while for younger children or those with bodyweight less than 30 kg, 10-15 ml/kg body weight is the recommended dose [17].

FFP contains all the coagulation factors and is mainly given in bleeding patients with prolonged prothrombin time (PT) and activated partial thromboplastin time (APTT) (more than 1.5 times the normal upper limit) at a dose of 10-20 ml/kg over 30 minutes with strict monitoring of hemodynamic status to avoid fluid overload (as DIC patients may require multiple blood component transfusions). In addition, PT and APTT normal values may vary in neonates with gestational age [2,18]. So, FFP transfusion should not be done based on coagulation parameters alone but should be considered in those with bleeding [1,2,18]. Adverse effects of FFP transfusion include viral infections (e.g. human immunodeficiency virus, hepatitis B, hepatitis C virus, and parvovirus B19), allergic reaction (urticaria, rarely anaphylaxis), transfusion-associated lung injury (higher incidence with FFP transfusions compared with platelet or packed red cell transfusions), and transfusion-associated cardiac overload [1].

Prothrombin complex concentrate (PCC) contains the vitamin K-dependent factors (II, VII, IX, and X), and can be considered in patients with bleeding if FFP cannot be transfused because of volume overload. However, it should be used judiciously as PCC can cause thrombosis and lacks certain coagulation factors (especially factor V) while in DIC, there is a global deficiency of coagulation factors [1,2]. To date, there is no evidence-based data on efficacy, safety, and optimal dosing of PCC in the pediatric population [16].

Severe hypofibrinogenemia (value less than 1.5 g/l with bleeding or less than 1 g/l that persists despite FFP replacement) may be treated with fibrinogen concentrate or cryoprecipitate [1,16]. Although there is a lack of clear evidence, the two may be considered equivocal alternatives. The recommended dose of fibrinogen concentrate is 50 ml/kg, and that of cryoprecipitate is 5-10 ml/kg [16,19]. Cryoprecipitate contains factor VIII, factor XIII, von Willebrand factor, fibrinogen, and fibronectin [19].

Anticoagulant - heparin

Few small-sized studies in the past (more than 30 years ago) revealed that therapeutic treatment with heparin in children with acute DIC had no mortality benefit, but it significantly improved the coagulation parameters [20,21]. Some studies in the 1970s have also clearly documented the undoubted therapeutic effectiveness of heparin in purpura fulminans and Waterhouse-Friderichsen syndrome following meningococcemia [22,23].

Therapeutic doses of heparin should be considered in those cases with predominant arterial or venous thromboembolism. However, if there is coexisting high risk of bleeding, continuous infusion of unfractionated heparin (UFH) can be considered, especially in the pediatric population since UFH has a short half-life and can be reversed with protamine sulfate. Monitoring of APTT may be complicated in these circumstances, and patients should be monitored for clinical signs of bleeding and anti-factor Xa levels [1,24]. Evidence is still lacking on the use of low molecular weight heparin (LMWH) in children with acute DIC. However, the prophylactic dose of LMWH is frequently used as supportive therapy for DIC in children with acute leukemia (heparin use in pediatric cancer patients is discussed separately) [3]. Since DIC is also associated with multiorgan failure, abnormal renal function can also complicate the treatment with LMWH [2].

Furthermore, the use of heparin for prophylaxis of DIC in neonates is probably restricted to those with obvious clinical signs of thromboembolism or prophylaxis in cases of indwelling central vascular catheters as there is an increased risk of bleeding complications in newborns [11]. Further studies are needed focusing on pediatric patients to establish therapeutic benefits of heparin in children with DIC.

Antithrombin

There are no randomized control trials available assessing the efficacy of antithrombin (AT) treatment in the pediatric population, but several retrospective analyses have reported its beneficial use [25-29]. Fuse et al. reported a case series in 1996 including four children with DIC and organ failure that were successfully treated with only AT concentrate [25].

Nowak-Göttl et al. used AT III without heparin in 21 preterm infants and 18 children with sepsis-induced DIC and found that there was normalization of plasma level of AT III, platelet count, fibrinogen, PT, APTT, and thrombin time within 24-48 hours without any observed side effects or mortality [26].

A multicenter post-marketing survey in 65 children with DIC concluded that AT replacement and concomitant anticoagulant therapy were safe and effective for DIC treatment when started at JMHW DIC score 6 or JAAM DIC score 4. The 28-day mortality rate was 6.8%, the standardized mortality rate was 0.55, and the DIC resolution rate on day three was 54.5%. In their study, AT replacement was started at AT activity 70% and DIC score of six, at a median total dose of 85.3 U/kg (median single dose of AT concentrate 30 U/kg) for a median duration of three days. Their study also concluded a target AT activity of 90% of normal at three days of therapy [27].

Another non-randomized multi-institutional prospective survey in 729 DIC patients (including 182 patients in the 15-64 years age group and two in <14 years age group) found that higher initial AT activity, AT supplementation dose at 3000 IU/day, and younger age were significant factors for improved survival, and the AT supplementation influenced both morbidity and mortality associated with severe sepsis. The risk of major bleeding was less than 2%, unaffected by concomitant heparin administration [28]. The authors also conducted a similar survey in a similar age group in 2014 and concluded that 3000 IU/day for three days was a safer and more efficacious dosing option than 1500 IU/day [29].

Based on these limited findings, AT supplementation and concomitant anticoagulation therapy may be considered safe for early DIC treatment in children. However, large-sized controlled studies have to be done to prove the efficacy and study the pharmacokinetics of AT supplementation in pediatric patients.

Activated protein C and protein C concentrate

Activated protein C (APC) inactivates the activated coagulation factors V and VIII, and ultimately causes inhibition of thrombin formation. It also has anti-inflammatory properties. So, it can play an important role in anti-DIC medicine as DIC involves activating the coagulation cascade and inflammatory processes [30].

There are two different formulations of protein C (PC) available: recombinant human activated protein C (rhAPC) and human plasma-derived viral inactivated protein C. During the neonatal period, since there is a higher risk of bleeding, the human plasma-derived viral-inactivated protein C concentrate may be an effective therapeutic option [31].

A prospective open-label study was done on eight children and adolescents with DIC associated with meningococcal septic shock and severe acquired protein C deficiency. They used virus-inactivated PrCC at the dose of 80 to 120 IU/kg i.v. bolus followed by 50 IU/kg up to six times per day. It resulted in normalization of plasma PC levels, marked decrement in plasminogen activator inhibitor type 1 levels, and significant clinical improvement without any adverse effects. Two out of eight patients died, and both had severely low plasma PC activity on admission [32].

Decembrino et al. conducted a pilot study in 18 neonates (12 preterm and six full-term) with severe sepsis or septic shock, with coagulopathy, and were given PrCC at the dose of 100 IU/kg i.v bolus, followed by 50 IU/kg every six hours for three days. Clinical Risk Index for Babies II score calculated the expected mortality in preterm babies as 10%. After 24 hours of treatment, PC activity levels increased from an average of 19% to 57% and were ultimately normalized in three days. The therapy also led to shortening of PT (P = 0.04) and APTT (P = 0.02), and an increase in AT levels (P < 0.0001), along with a reduction in C-reactive protein (P = 0.005) and Neonatal Therapeutic Intervention Scoring System values (P = 0.003). There were no adverse reactions and no mortality [33].

Drotrecogin alfa is a rhAPC used for the treatment of adults with severe sepsis but a multicenter phase III randomized controlled trial called Researching Severe Sepsis and Organ Dysfunction in Children: A Global Perspective (RESOLVE) trial concluded that there is no efficacy of its use in children with severe sepsis [34]. In addition, a systematic review by Kylat and Ohlsson in 2012 also concluded that there is insufficient data to use rhAPC to manage severe sepsis in newborn infants. Results among adults demonstrated a lack of efficacy and increase in bleeding, ultimately resulting in the withdrawal of rhAPC from the market. So, the authors concluded that neonates should not be treated with rhAPC, and further trials should not be conducted [35].

Based on these findings, protein C concentrate (PrCC) can be considered as a beneficial therapeutic option, but controlled studies have to be done to prove the efficacy. However, rhAPC is not recommended for use in pediatric patients.

Recombinant thrombomodulin

Studies in the past two decades have established thrombomodulin to be a crucial component of a multimolecular system with antithrombotic, anti-inflammatory, and cytoprotective properties. rTM activates protein C leading to the inactivation of factor Va, which ultimately leads to the inhibition of thrombin generation. This results in minimal effects on clotting times compared to the effects of argatroban, heparin, or recombinant APC. Along with this, there is also a lower risk of bleeding with rTM than APC [36,37]. Few studies on rTM therapy are summarized in Table 3 [9,36,38].

**Table 3:** Summary of findings from studies done by Yagasaki et al., Shirahata et al., and Mimuro et al. on rTM therapy on the management of DIC in children. AT, anti-thrombin; DIC, disseminated intravascular coagulation; FDP, fibrin degradation products; FFP, fresh frozen plasma; rTM, recombinant thrombomodulin; UFH, unfractionated heparin; RBC, red blood cells; LMWH, low molecular weight heparin; ADR, adverse drug reactions.

rTM therapy	Yagasaki et al. (2012) [9]	Shirahata et al. (2014) [36]	Mimuro et al. (2013) [38]
Type of study	Retrospective study	Post-marketing surveillance	Post-marketing surveillance
Age group and number of cases	Children: 25	Neonates: 60	0-14 years: 270; 15-64 years: 1484; >65 years: 2302; total: 3548
Daily dose	380 U/kg/day i.v. over 30 min	380 U/kg/day i.v. over 30 min	380 U/kg/day (0.06 mg/kg) i.v. over 30 min
Dose for severe renal impairment	130 U/kg/day, given to newborns considering their relatively low renal function	130 U/kg/day	130 U/kg/day
Duration	Median duration five days (range 2-13 days)	Less than six days because of earlier improvement in 59.4% of cases; for six days in 15.8% of cases, with no change in DIC; for more than six days in 31.7% of cases	28 days
Concomitant drugs	FFP and platelets; one patient had concomitant AT with rTM	rTM monotherapy in 31.7%; concomitant anticoagulants in 68.3%; AT concentrates, nafamostat mesylate, UFH, and gabexate mesylate; concomitant blood products usage: platelet concentrate, FFP, and RBC concentrate	Concomitant anticoagulants in 36.8%: gabexate mesylate, AT concentrates, nafamostat mesylate, UFH, heparan sulfate, and LMWH
Outcome	Survival rate at day 28 was 22/25; in seven days, 20/25 patients recovered from DIC; had significant improvement in Median Pediatric Logistic Organ Dysfunction score and in the values of prothrombin time ratio, fibrin/ FDP, D-dimer, and protein C; 5/25 patients failed to respond	Survival rate at day 28: 76.7%; DIC resolution rate: 47.1%; decreased levels of fibrin/FDP, increased platelet counts, and AT activity​​​​​​​; DIC improvement rate and survival rates were greater in neonatal patients than in children and adults, but the DIC resolution rate was lower	Survival rate at day 28: in infection-induced DIC, 64.1%; in hematological malignancy-associated DIC, 70.7%;​​​​​​​ DIC scores significantly decreased in both groups ( P-value < 0.001);​​​​​​​ severity of underlying disease was the most important factor for survival rate (odds ratio: 0.288, P < 0.001)
Adverse drug reactions (ADR)	Serious bleeding in two out of 25 children	ADR: 6.7%; bleeding-related ADR: 6.7%;​​​​​​​ bleeding-related adverse events: 16.7 %	Critical bleeding ADR in the infection-DIC: 2.6% and hemat-DIC groups: 2.4%; younger age and pre-existing bleeding were found to affect the bleeding ADR

Compared to other anticoagulants, rTM seems to be more effective and safe in children. However, further trials are needed to confirm its efficacy.

Antifibrinolytics

Antifibrinolytic agents and recombinant FVIIa are not recommended in the treatment of DIC due to significant concern for thromboembolism except in patients with hyperfibrinolysis, such as acute promyelocytic leukemia [2,39].

Tissue factor pathway inhibitor

In sepsis-induced DIC, tissue factor (TF) and factor VIIa pathway mainly activate coagulation cascade and increase TF expression compared to tissue factor pathway inhibitor (TFPI). So, replacement therapy with recombinant TFPI is a rational therapeutic approach [40]. Although there have been a few randomized controlled trials in adults, no such studies have been done in pediatrics.

Others 

Few case series and reports have reported using plasma exchange and newer therapeutic approaches like eculizumab, but no proven beneficial role has been established [41,42].

Combination therapy

A prospective interventional and open-labeled randomized controlled trial was done recently in 2021 in 80 PICU patients with proven severe sepsis/septic shock and non-overt DIC. The intervention group was given FFP, low-dose UFH, and tranexamic acid. Compared with this group, the non-intervention group had a significantly higher mortality rate, a significantly higher rate of progression to overt DIC (45% vs 10%, P < 0.0001), and significantly higher DIC risk assessment scores on the second and fifth day [43].

A retrospective cohort study was done in 55 neonates with DIC. Group 1 received (FFP) or (FFP + AT III), while Group 2 received (rTM) or (rTM + FFP + AT III). They found that the DIC scores were reduced in both groups on day one and day two with P-value < 0.05. Furthermore, DIC scores before treatment in Group 2 were higher than in Group 1 (4.7 vs 3.6, P < 0.05). Hence, they concluded that rTM and FFP were effective therapies for neonatal DIC [44].

A randomized prospective, double-blind trial in 2002 included patients more than 15 years of age and found that APC in a relatively small dosage (dosage used: APC at 2.5 g/kg/hr and heparin at 8 U/kg/hr by intravenous infusion) can improve DIC more efficiently than can heparin. The use of APC did not increase bleeding, and this trial suggested that APC may be a better alternative [45].

Treatment options for DIC in pediatric cancer

There is no evidence-based guideline regarding the management of DIC in children with cancer. So, general DIC treatment guidelines are followed: platelet concentrate transfusion in bleeding patients with platelet count <50,000/μL and FFP transfusion in those with active bleeding and PT, and APTT more than 1.5 times the normal upper limit. However, in non-bleeding patients who develop DIC during chemotherapy, a threshold platelet count of <10,000/μL is also accepted. The use of heparin and antifibrinolytics like tranexamic acid and epsilon aminocaproic acid is still controversial in pediatric cancer patients [3].

A systematic review of 24 articles done by Kongstad et al. in 2020 found that a prophylactic dose of unfractionated heparin was most frequently used as a supportive agent in pediatric acute leukemia. However, most of the children developed hemorrhages leading to death in the studies, and also, the articles were almost 30 years old. So, they considered heparin treatment contradictory [3]. Their findings are included in Table 4.

**Table 4:** Supportive therapy used for DIC in pediatric leukemia in different articles. Adapted from Kongstad et al. [3].

Supportive therapy used	No. of articles
Unfractionated heparin	9
Platelet concentrate	8
Fresh frozen plasma	5
Cryoprecipitate	2
Epsilon aminocaproic acid	2
Vitamin K	1
Tranexamic acid	1
Recombinant thrombomodulin	1

A randomized controlled trial in 60 children with acute leukemia demonstrated that bleeding tendency and DIC incidence were greatly decreased with an early intervention using low molecular weight heparin before DIC occurrence [46].

**Figure 1:**
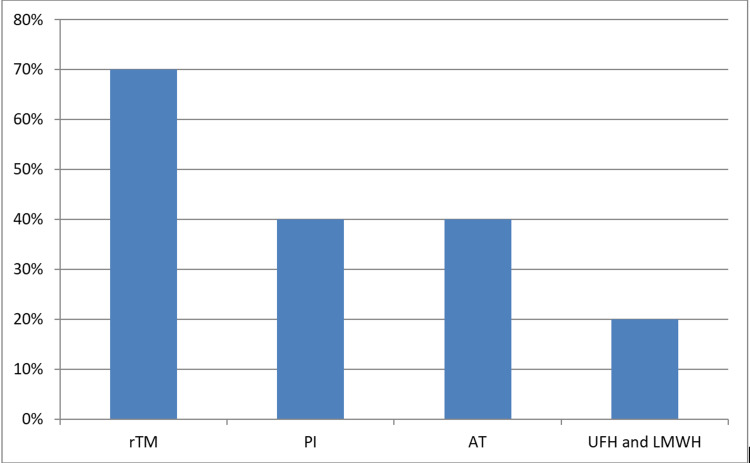
Selection of first-line anticoagulant therapy for DIC in pediatric leukemia in Japan. Adapted from Osone et al. [47]. AT, antithrombin concentrate; LMWH, low molecular weight heparin; PI, synthetic protease inhibitors (gabexate mesylate, nafamostat mesylate); rTM, recombinant thrombomodulin; UFH, unfractionated heparin.

A national survey regarding the supportive treatment of hemostatic complications in children with leukemia was done by Osone et al. in Japan in 2019, and they found that the management differs markedly from protocols in other countries. rTM was most often used in Japan irrespective of the etiology of DIC. Other treatment options and their findings are summarized in Figure 1. They also found that dalteparin and danaparoid sodium were used rather than enoxaparin for LMWH (dalteparin more than danaparoid) [47].

Osone et al. also studied the use of supportive therapy for hemostatic alterations caused by asparaginase as induction therapy in childhood leukemia [47]. Prophylactic antithrombin replacement in such conditions was used by 95% of institutions and most of them used a threshold of <70 % AT activity (67%); while some used <60% or <50% of AT activity as a threshold. Prophylactic FFP transfusion, however, was used by only 24% of total institutions in the study, and 58% of them used a threshold of <0.5 g/l, and 18% used <1 g/l [47].

A retrospective study of adults by Ookura et al. in 2018 compared the efficacy of rTM with gabexate mesylate (GM) and concluded that rTM is safe and more efficacious than GM for treating acute myeloid leukemia patients with DIC [48].

There are only limited moderate quality and heterogeneous studies in pediatric cancer patients. The use of heparin, antifibrinolytics, protease inhibitors, or replacement therapy with blood components is still based on clinical judgments and findings in the adult population. rTM is emerging as new beneficial therapy, but high-quality large-sized studies are yet to be done.

Limitations

This article focuses on the pediatric population, but one article under the subheading "Anticoagulant - heparin" involves more than 15 years age group, two articles under the subheading "Antithrombin" and one under "Recombinant thrombomodulin" involves both pediatric and adult age groups. The studies were included because only a few studies involved the pediatric age groups under the respective subheadings and the findings were significant. Large-sized studies were limited, so most of the studies included are small or moderate-sized.

The article involves studies from 1990 to 2021, but four articles under the subheading "Anticoagulant - heparin" were published before 1990. The articles were included because no recent studies were found during our database search.

## Conclusions

High-quality studies in disseminated intravascular coagulation (DIC) diagnosis or management are limited in the pediatric population. Current practices mainly depend on clinical experiences, expert opinions, and findings based on the adults. Limited studies have shown that different diagnostic scoring systems used in adults work fairly well in pediatric patients, but their efficacy has not been validated. Decisions on diagnosis, severity, or course of DIC should not be based on a single, one-time DIC score, but rather sequential evaluation of clinical and laboratory parameters is recommended. Treatment of underlying disease is the cornerstone of DIC management. Replacement therapy with blood products such as fresh frozen plasma, platelet transfusion, cryoprecipitate, or fibrinogen concentrate should not be primarily based on laboratory derangements, but should only be done in patients with bleeding manifestations. Good-quality evidence for transfusion of the non-red blood cell products in the pediatric population is low. Limited evidence suggests that supplementation with antithrombin, protein C concentrate, or recombinant thrombomodulin is beneficial for DIC treatment in children. However, larger randomized controlled studies are needed to prove efficacy.

## References

[1] Levi M, Toh CH, Thachil J, Watson HG (2009). Guidelines for the diagnosis and management of disseminated intravascular coagulation. British Committee for Standards in Haematology. Br J Haematol.

[2] Rajagopal R, Thachil J, Monagle P (2017). Disseminated intravascular coagulation in paediatrics. Arch Dis Child.

[3] Kongstad C, Mikkelsen TS, Hvas AM (2020). Disseminated intravascular coagulation in children with cancer: a systematic review. Pediatr Hematol Oncol.

[4] Toh CH, Hoots WK (2007). The scoring system of the Scientific and Standardisation Committee on Disseminated Intravascular Coagulation of the International Society on Thrombosis and Haemostasis: a 5-year overview. J Thromb Haemost.

[5] Gando S (2012). The utility of a diagnostic scoring system for disseminated intravascular coagulation. Crit Care Clin.

[6] Khemani RG, Bart RD, Alonzo TA, Hatzakis G, Hallam D, Newth CJ (2009). Disseminated intravascular coagulation score is associated with mortality for children with shock. Intensive Care Med.

[7] Soundar EP, Jariwala P, Nguyen TC, Eldin KW, Teruya J (2013). Evaluation of the International Society on Thrombosis and Haemostasis and institutional diagnostic criteria of disseminated intravascular coagulation in pediatric patients. Am J Clin Pathol.

[8] Jhang WK, Ha EJ, Park SJ (2016). Evaluation of disseminated intravascular coagulation scores in critically ill pediatric patients. Pediatr Crit Care Med.

[9] Yagasaki H, Kato M, Shimozawa K (2012). Treatment responses for disseminated intravascular coagulation in 25 children treated with recombinant thrombomodulin: a single institution experience. Thromb Res.

[10] Veldman A, Fischer D, Nold MF, Wong FY (2010). Disseminated intravascular coagulation in term and preterm neonates. Semin Thromb Hemost.

[11] Rhodes A, Evans LE, Alhazzani W (2017). Surviving sepsis campaign: international guidelines for management of sepsis and septic shock: 2016. Intensive Care Med.

[12] Wada H, Matsumoto T, Hatada T (2012). Diagnostic criteria and laboratory tests for disseminated intravascular coagulation. Expert Rev Hematol.

[13] Davis AL, Carcillo JA, Aneja RK (2017). American College of Critical Care Medicine clinical practice parameters for hemodynamic support of pediatric and neonatal septic shock. Crit Care Med.

[14] Thachil J, Toh CH (2012). Current concepts in the management of disseminated intravascular coagulation. Thromb Res.

[15] Patel RM, Josephson C (2019). Neonatal and pediatric platelet transfusions: current concepts and controversies. Curr Opin Hematol.

[16] Steinbicker AU, Wittenmeier E, Goobie SM (2020). Pediatric non-red cell blood product transfusion practices: what's the evidence to guide transfusion of the 'yellow' blood products?. Curr Opin Anaesthesiol.

[17] Sloan SR, Parker RI (2016). Current status of platelet transfusion in pediatric patients. Transfus Med Rev.

[18] Pal S, Curley A, Stanworth SJ (2015). Interpretation of clotting tests in the neonate. Arch Dis Child Fetal Neonatal Ed.

[19] Kelly AM, Williamson LM (2013). Neonatal transfusion. Early Hum Dev.

[20] Corrigan JJ Jr, Jordan CM (1970). Heparin therapy in septicemia with disseminated intravascular coagulation. N Engl J Med.

[21] Göbel U, von Voss H, Jürgens H, Petrich C, Pothmann R, Sprock I, Lemburg P (1980). Efficiency of heparin in the treatment of newborn infants with respiratory distress syndrome and disseminated intravascular coagulation. Eur J Pediatr.

[22] Lo SS, Hitzig WH, Frick PG (1971). Clinical experience with anticoagulant therapy in the management of disseminated intravascular coagulation in children. Acta Haematol.

[23] Fox B (1971). Disseminated intravascular coagulation and the Waterhouse-Friderichsen syndrome. Arch Dis Child.

[24] Hanslik A, Kitzmüller E, Tran US (2015). Monitoring unfractionated heparin in children: a parallel-cohort randomized controlled trial comparing 2 dose protocols. Blood.

[25] Fuse S, Tomita H, Yoshida M, Hori T, Igarashi C, Fujita S (1996). High dose of intravenous antithrombin III without heparin in the treatment of disseminated intravascular coagulation and organ failure in four children. Am J Hematol.

[26] Nowak-Göttl U, Groll A, Kreuz WD, Brand M, Breddin HK, von Loewenich V, Kornhuber B (1992). Treatment of disseminated intravascular coagulation with antithrombin III concentrate in children with verified infection. (Article in German). Klin Padiatr.

[27] Nagafuchi H, Eguchi Y, Ikeda T (2019). Impact of antithrombin supplementation and concomitant anticoagulation therapy in pediatric patients with disseminated intravascular coagulation. Clin Appl Thromb Hemost.

[28] Iba T, Saito D, Wada H, Asakura H (2012). Efficacy and bleeding risk of antithrombin supplementation in septic disseminated intravascular coagulation: a prospective multicenter survey. Thromb Res.

[29] Iba T, Saitoh D, Wada H, Asakura H (2014). Efficacy and bleeding risk of antithrombin supplementation in septic disseminated intravascular coagulation: a secondary survey. Crit Care.

[30] Maruyama I (1999). Recombinant thrombomodulin and activated protein C in the treatment of disseminated intravascular coagulation. Thromb Haemost.

[31] De Carolis MP (2010). Use of protein C concentrate in neonatal period. Minerva Pediatr.

[32] Ettingshausen CE, Veldmann A, Beeg T, Schneider W, Jäger G, Kreuz W (1999). Replacement therapy with protein C concentrate in infants and adolescents with meningococcal sepsis and purpura fulminans. Semin Thromb Hemost.

[33] Decembrino L, D'Angelo A, Manzato F (2010). Protein C concentrate as adjuvant treatment in neonates with sepsis-induced coagulopathy: a pilot study. Shock.

[34] Nadel S, Goldstein B, Williams MD (2007). Drotrecogin alfa (activated) in children with severe sepsis: a multicentre phase III randomised controlled trial. Lancet.

[35] Kylat RI, Ohlsson A (2012). Recombinant human activated protein C for severe sepsis in neonates. Cochrane Database Syst Rev.

[36] Shirahata A, Mimuro J, Takahashi H (2014). Recombinant soluble human thrombomodulin (thrombomodulin alfa) in the treatment of neonatal disseminated intravascular coagulation. Eur J Pediatr.

[37] Ito T, Thachil J, Asakura H, Levy JH, Iba T (2019). Thrombomodulin in disseminated intravascular coagulation and other critical conditions-a multi-faceted anticoagulant protein with therapeutic potential. Crit Care.

[38] Mimuro J, Takahashi H, Kitajima I (2013). Impact of recombinant soluble thrombomodulin (thrombomodulin alfa) on disseminated intravascular coagulation. Thromb Res.

[39] Yang H, Zhu CY, Wang QS (2014). Analysis of empirical treatment for newly diagnosed acute promyelocytic leukemia combined with disseminated intravascular coagulation. (Article in Chinese). Zhongguo Shi Yan Xue Ye Xue Za Zhi.

[40] Papageorgiou C, Jourdi G, Adjambri E (2018). Disseminated intravascular coagulation: an update on pathogenesis, diagnosis, and therapeutic strategies. Clin Appl Thromb Hemost.

[41] Galic S, Csuka D, Prohászka Z, Turudic D, Dzepina P, Milosevic D (2019). A case report of a child with sepsis induced multiorgan failure and massive complement consumption treated with a short course of eculizumab: a case of crosstalk between coagulation and complement?. Medicine (Baltimore).

[42] Keir AK, Stanworth SJ (2016). Neonatal plasma transfusion: an evidence-based review. Transfus Med Rev.

[43] El-Nawawy AA, Elshinawy MI, Khater DM, Moustafa AA, Hassanein NM, Wali YA, Nazir HF (2021). Outcome of early hemostatic intervention in children with sepsis and nonovert disseminated intravascular coagulation admitted to PICU: a randomized controlled trial. Pediatr Crit Care Med.

[44] Go H, Ohto H, Nollet KE (2020). Risk factors and treatments for disseminated intravascular coagulation in neonates. Ital J Pediatr.

[45] Aoki N, Matsuda T, Saito H (2002). A comparative double-blind randomized trial of activated protein C and unfractionated heparin in the treatment of disseminated intravascular coagulation. Int J Hematol.

[46] Yun-Peng G, Li-Rong S, Yan-Xia Z, Ren Z (2008). Effect of early intervention using low molecular weight heparin in DIC in children with acute leukemia. J Clin Pediatr.

[47] Osone S, Fukushima K, Yano M (2019). Supportive care for hemostatic complications associated with pediatric leukemia: a national survey in Japan. Int J Hematol.

[48] Ookura M, Hosono N, Tasaki T (2018). Successful treatment of disseminated intravascular coagulation by recombinant human soluble thrombomodulin in patients with acute myeloid leukemia. Medicine (Baltimore).

